# Reported Life-Threatening Polymorphic Ventricular Arrhythmia With Torsade-Like Morphology in a Patient With Gordon Syndrome and Hyperkalaemia

**DOI:** 10.7759/cureus.117322

**Published:** 2026-10-01

**Authors:** Laiba Kayani, Ayman Emam, Rithima Nair, Saba Elmahadi

**Affiliations:** 1 Trauma and Orthopaedics, Kings Mill Hospital, Mansfield, GBR; 2 Cardiology, Nottingham City Hospital, Nottingham, GBR

**Keywords:** cul3 mutation, extravascular implantable cardioverter-defibrillator, gordon syndrome, hyperkalemia, medication adherence, polymorphic ventricular tachycardia, pseudohypoaldosteronism type ii, thiazide therapy, torsade de pointes

## Abstract

Gordon's syndrome (pseudohypoaldosteronism type II) is a rare, inherited tubulopathy characterised by salt-sensitive hypertension, hyperkalaemia, and metabolic acidosis despite preserved renal function. Pathogenic variants affecting the with-no-lysine (K) signalling pathway include WNK1, WNK4, KLHL3, and CUL3.

We report the case of a 37-year-old woman with genetically confirmed CUL3-related Gordon's syndrome who developed hyperkalaemia after a three-week interruption of bendroflumethiazide. During pre-hospital assessment, ambulance clinicians documented a self-terminating rhythm that they identified as torsade de pointes. The original recording cannot be retrieved; consequently, the rhythm classification, preceding QT interval, and possible alternative rhythm or artefact could not be independently assessed.

Point-of-care venous blood gas potassium was 4.8 mmol/L, whereas formal laboratory potassium was 6.1 mmol/L. The sampling and processing information available did not establish the cause of this discrepancy. Subsequent echocardiography, computed tomography coronary angiography, cardiac magnetic resonance imaging, ambulatory monitoring, exercise testing, and additional electrical assessment did not identify a definitive alternative substrate, although an intermittent electrical disorder could not be excluded.

Following treatment of hyperkalaemia and multidisciplinary cardiology and electrophysiology review, an extravascular implantable cardioverter-defibrillator was implanted. Bendroflumethiazide was resumed, with subsequent improvement in potassium control. This case highlights the importance of uninterrupted thiazide therapy in Gordon's syndrome, assessment of discordant potassium measurements, and careful interpretation of a reported serious rhythm event when its original tracing is unavailable. The temporal association with hyperkalaemia does not establish that it caused torsade de pointes.

## Introduction

Gordon's syndrome (pseudohypoaldosteronism type II) is a rare, inherited tubulopathy characterised by salt-sensitive hypertension, hyperkalaemia, and hyperchloraemic metabolic acidosis despite preserved renal function [1]. It results from abnormalities in the with-no-lysine (K) or WNK signalling pathway, with pathogenic variants identified in WNK1, WNK4, KLHL3, and CUL3 [1,2]. CUL3-related disease typically has an earlier and more severe phenotype [1]. Thiazide diuretics target the increased activity of the sodium-chloride cotransporter associated with this pathway and are important for controlling both blood pressure and potassium in Gordon's syndrome [1,2].

Torsade de pointes is a form of polymorphic ventricular tachycardia associated with QT prolongation and may be precipitated by hypokalaemia or hypomagnesaemia [3]. Hyperkalaemia more typically causes peaked T waves, conduction slowing, QRS widening, and, at extreme concentrations, a sine-wave pattern. We report the case of a 37-year-old woman with CUL3-related Gordon's syndrome who developed hyperkalaemia after a 3-week interruption of bendroflumethiazide therapy. During pre-hospital assessment, ambulance clinicians documented a self-terminating rhythm that they identified as torsade de pointes. Because the original recording cannot be retrieved, the rhythm classification and its relationship to hyperkalaemia cannot be independently established.

## Case presentation

A 37-year-old woman with genetically confirmed CUL3-related Gordon's syndrome and Crohn's disease presented to the emergency department after an episode of central chest pain and severe dizziness at home.

The pain began suddenly while she was cooking. It was sharp, radiated towards the left shoulder, back, and abdomen, and was accompanied by dizziness on standing, dyspnoea, and nausea. The ambulance report describes the pain as lasting approximately one hour and resolving before assessment by the crew. Her initial recorded blood pressure was 158/92 mmHg.

The patient had been unable to take her regular bendroflumethiazide 2.5 mg once daily for approximately three weeks while awaiting primary care review and re-prescribing. Earlier clinical records documented marked hyperkalaemia requiring repeated insulin and dextrose infusions during a previous prolonged interruption of bendroflumethiazide.

The ambulance crew documented sinus arrhythmia on their initial 3-lead and 12-lead ECG assessment. After the patient was reconnected to three-lead monitoring in the ambulance, the crew documented an episode they identified as torsade de pointes. Their contemporaneous report states that she remained alert and that the episode resolved without defibrillation, chest compressions, or medication. Defibrillator pads were applied, and she was transferred under cardiac monitoring.

The original recording of the episode cannot be retrieved. The available ambulance report does not state its duration or rate, whether a pulse was assessed during the episode, the immediately preceding QT interval, or the rhythm features on which the classification was based. The strong radial pulse documented during the crew's initial assessment was recorded before the reported rhythm episode. The rhythm diagnosis therefore cannot be independently verified, and an alternative rhythm or monitoring artefact cannot be excluded.

The clinical course is summarised in Figure 1.

**Figure 1:**
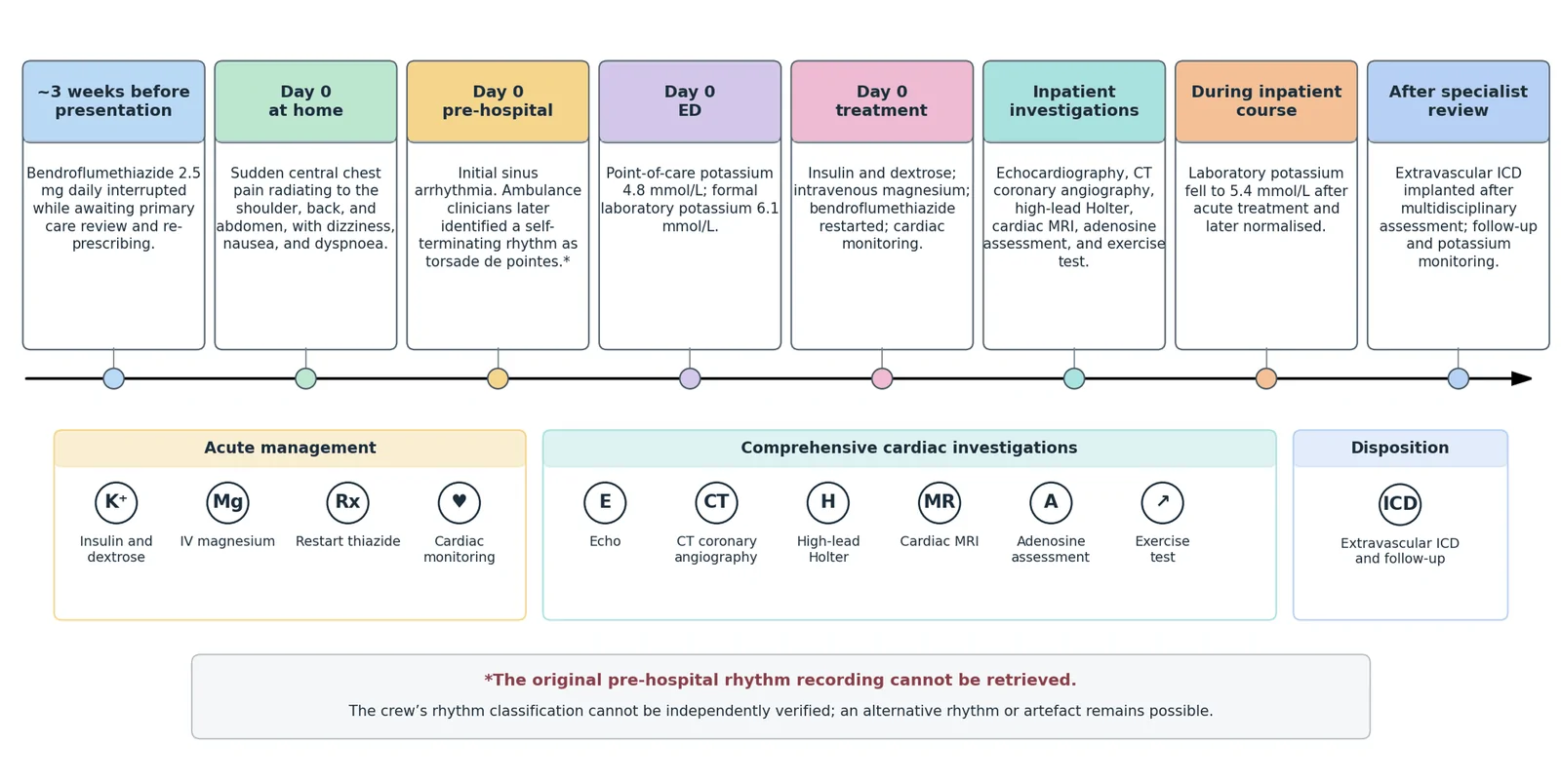
Clinical timeline of presentation, investigation, and management Timeline from interruption of bendroflumethiazide to the pre-hospital rhythm episode, emergency treatment, inpatient cardiac investigations, and extravascular implantable cardioverter-defibrillator implantation. Ambulance clinicians identified the self-terminating rhythm as torsade de pointes, but the original recording cannot be retrieved for independent review. The figure therefore does not establish the rhythm classification or a causal relationship with hyperkalaemia. ED: emergency department; CT: computed tomography; MRI: magnetic resonance imaging; ICD: implantable cardioverter-defibrillator

Point-of-care venous blood gas analysis recorded potassium of 4.8 mmol/L, whereas formal laboratory analysis recorded potassium of 6.1 mmol/L (serum reference range: 3.5-5.3 mmol/L). The available records do not establish whether the two samples were collected simultaneously or from the same venepuncture, their precise interval, or whether either sample was affected by haemolysis. The 1.3 mmol/L difference therefore cannot be assigned a specific cause. Repeat laboratory potassium later on the day of presentation was 5.4 mmol/L after acute treatment (Table 1).

**Table 1:** Serial potassium measurements The formal laboratory serum reference range was 3.5-5.3 mmol/L, and the point-of-care venous blood gas reference range was 3.4-4.5 mmol/L. The precise sampling interval, whether the initial samples came from the same venepuncture, and their haemolysis status could not be confirmed from the available records.

Time point	Potassium (mmol/L)	Method and context
Day 0, initial emergency department assessment	4.8	Point-of-care venous blood gas
Day 0, admission	6.1	Formal laboratory analysis
Day 0, after acute treatment	5.4	Formal laboratory analysis
Subsequent inpatient course	Normalised	Following treatment and resumption of bendroflumethiazide

Admission magnesium was 0.8 mmol/L (reference range: 0.7-1.0 mmol/L), sodium 137 mmol/L (133-146 mmol/L), urea 2.8 mmol/L (2.5-7.8 mmol/L), creatinine 48 µmol/L (adult female reference range: 49-90 µmol/L), and C-reactive protein <1 mg/L (reference range: <5 mg/L). Cardiac troponin I was <1.6 ng/L (adult female 99th percentile: <15 ng/L). Acid-base measurements sufficient to characterise the patient's status at the time of the reported rhythm episode were not available in the records reviewed.

Hyperkalaemia was treated with insulin and dextrose, and intravenous magnesium sulphate was administered following the reported rhythm episode. Bendroflumethiazide 2.5 mg once daily was restarted. Propranolol was commenced during inpatient investigation and was subsequently held before exercise testing. The available information does not establish whether it was continued after discharge. Cardiac monitoring was maintained during admission.

Cardiac investigations were undertaken to assess possible structural, coronary, and electrical causes of the reported event (Table 2). Transthoracic echocardiography showed a non-dilated left ventricle with normal wall thickness and diastolic function and a visually estimated ejection fraction greater than 55%. Right ventricular systolic function was normal, with no significant valvular abnormality.

**Table 2:** Summary of cardiac investigations Findings obtained after the reported pre-hospital event do not independently verify its rhythm or exclude every intermittent electrical disorder.

Investigation	Main findings and limits
Pre-hospital ECG monitoring	Initial sinus arrhythmia; ambulance clinicians subsequently documented a self-terminating episode they identified as torsade de pointes on three-lead monitoring. The original episode recording cannot be retrieved, and the rhythm cannot be independently classified.
Transthoracic echocardiography	Non-dilated left ventricle with normal wall thickness and diastolic function; visually estimated ejection fraction >55%; normal right ventricular systolic function; no significant valvular abnormality.
CT coronary angiography	Conventional right-dominant coronary anatomy; no evidence of coronary artery disease. This does not exclude transient vasospasm.
24-hour 12-lead Holter monitoring	V1 and V2 placed in the second intercostal space; sinus rhythm at 53–109 beats/min, mean 73 beats/min; 325 supraventricular ectopic beats (<1%); no ventricular ectopy or recurrent arrhythmia.
Cardiac MRI	Normal biventricular volumes and function; left ventricular ejection fraction 69% and right ventricular ejection fraction 70%; raised inferior septal and inferior wall T1 values (1239–1400 ms); no convincing oedema; possible subtle inferior mid-wall enhancement without definite scar.
Adenosine assessment	Transient atrioventricular nodal block without evidence of accessory pathway conduction.
Exercise stress testing	No significant ST-segment changes, T-wave abnormalities, or ventricular arrhythmias.

CT coronary angiography showed conventional right-dominant coronary anatomy and no evidence of coronary artery disease. The available documentation does not specify why CT coronary angiography was selected instead of invasive coronary angiography. A normal CT coronary angiogram does not, by itself, exclude transient coronary vasospasm.

A 24-hour 12-lead Holter recording was obtained with V1 and V2 placed in the second intercostal space. The report noted some artefacts but described sinus rhythm throughout, with a heart-rate range of 53-109 beats/min and an average of 73 beats/min. There were 325 supraventricular ectopic beats, representing less than 1% of recorded beats, and no ventricular ectopy or recurrent ventricular arrhythmia. This later recording cannot establish the nature of the earlier pre-hospital episode.

Cardiac MRI showed normal biventricular volumes and systolic function, with a left ventricular ejection fraction of 69% and right ventricular ejection fraction of 70%. Native T1 values were raised in the inferior septum and inferior wall (1239-1400 ms). Short tau inversion recovery imaging showed no convincing myocardial oedema. Possible subtle mid-wall enhancement in the mid-inferior wall was reported but was not considered conclusive evidence of scar. These findings were regarded as non-specific.

Intravenous adenosine 12 mg produced transient atrioventricular nodal block without evidence of accessory pathway conduction. Exercise stress testing did not demonstrate significant ST-segment changes, T-wave abnormalities, or ventricular arrhythmias. The available material does not provide a reliable QTc measurement from an ECG recorded during hyperkalaemia or immediately after the reported pre-hospital episode. No diagnostic Brugada provocation result or completed inherited-arrhythmia genetic test result was available for inclusion in this report.

The original genetic laboratory report identified a heterozygous pathogenic CUL3 splice-site variant, NM_003590.4.1377+1G>A (GRCh37, chr2.225368368C>T), confirming CUL3-related Gordon's syndrome. The report did not establish whether the variant was inherited or arose de novo. Earlier records noted no family history in relation to Gordon's syndrome, but the available material does not document a specific negative family history of sudden cardiac death. The CUL3 finding does not establish a separate inherited arrhythmia syndrome.

The differential diagnosis included an electrolyte-associated arrhythmia and an underlying electrical disorder. Hyperkalaemia following interruption of bendroflumethiazide was a plausible contributor, but its causal role and the classification of the reported rhythm cannot be established from the available evidence. The subsequent investigations did not identify a definitive alternative structural or electrical substrate; they cannot exclude all intermittent or inherited causes.

Following multidisciplinary cardiology and electrophysiology review, an extravascular ICD was implanted. This was an individual management decision made despite preserved ventricular function and a potentially reversible electrolyte disturbance, in the context of the reported pre-hospital event, uncertainty about its mechanism, and concern about recurrence of metabolic disturbance. The implantation was uncomplicated.

Potassium improved after acute treatment and resumption of bendroflumethiazide and remained stable during subsequent monitoring. The patient was counselled about the importance of uninterrupted thiazide therapy and continued under cardiology and specialist hypertension follow-up. Genetic counselling and screening of first-degree relatives were discussed.

## Discussion

Gordon's syndrome is an inherited disorder of electrolyte and blood-pressure regulation caused by abnormalities in the WNK signalling pathway. Pathogenic variants involving WNK1, WNK4, KLHL3, and CUL3 alter regulation of the thiazide-sensitive sodium-chloride cotransporter in the distal nephron. The resulting increase in sodium reabsorption contributes to hypertension, impaired potassium excretion, and hyperkalaemic metabolic acidosis [1,2,4].

The pre-hospital event was unusual because the attending ambulance clinicians identified the rhythm as torsade de pointes in a patient subsequently found to have hyperkalaemia. Classical torsade de pointes is associated with QT prolongation and may be precipitated by hypokalaemia or hypomagnesaemia [3]. Hyperkalaemia more commonly causes peaked T waves, conduction slowing, and QRS widening. The admission magnesium concentration of 0.8 mmol/L was within the laboratory reference range; hypomagnesaemia was not demonstrated.

The original pre-hospital recording cannot be retrieved, and the available report does not describe the rhythm's duration, rate, mode of initiation, or preceding QT interval. Its classification as torsade de pointes therefore cannot be independently verified. An alternative rhythm or monitoring artefact cannot be excluded. Remaining alert during a reported episode and recovering without intervention do not establish its mechanism. Similarly, subsequent investigations showing no recurrent ventricular arrhythmia cannot retrospectively confirm what occurred in the ambulance. We have consequently attributed the rhythm description to the attending clinicians throughout this report rather than presenting polymorphic ventricular tachycardia or classical torsade de pointes as independently established diagnoses.

The potassium concentration of 6.1 mmol/L following three weeks without bendroflumethiazide provides a plausible metabolic context for the reported event. Nevertheless, potassium was measured after the pre-hospital episode, no contemporaneous event tracing is available, and an ECG demonstrating the effects of hyperkalaemia at the time of the episode cannot be assessed. The temporal association does not establish that hyperkalaemia caused the reported rhythm. The patient's earlier marked hyperkalaemia during a separate interruption of thiazide therapy supports a recurring vulnerability to potassium elevation when treatment is stopped, but does not establish an arrhythmia mechanism in this presentation.

The difference between the point-of-care venous blood gas potassium of 4.8 mmol/L and formal laboratory potassium of 6.1 mmol/L was clinically important. Blood gas and laboratory potassium results can differ because they use different specimen types and analytical methods and may be affected by collection technique, processing, haemolysis, and the interval between sampling [5]. The available records do not establish whether these samples were taken simultaneously or from the same venepuncture, their precise sampling interval, or their haemolysis status. No specific cause of the discrepancy can therefore be identified. The subsequent formal laboratory result of 5.4 mmol/L was obtained after treatment and does not independently establish which initial measurement best represented the potassium concentration at the time of the pre-hospital event. This case supports promptly checking an unexpected point-of-care potassium result against formal laboratory testing while assessing the patient and ECG in context.

Thiazides target the underlying sodium-chloride cotransporter abnormality in Gordon's syndrome and are important for controlling both blood pressure and potassium [6]. The repeated deterioration in potassium control during interruptions of bendroflumethiazide in this patient highlights the importance of maintaining access to treatment.

The subsequent evaluation did not identify a definite alternative cardiac substrate. Echocardiography showed preserved ventricular function without left ventricular hypertrophy. CT coronary angiography demonstrated no coronary artery disease, although it cannot exclude a transient episode of coronary vasospasm. The available records do not document why CT coronary angiography was selected instead of invasive angiography or whether vasospasm was specifically considered by the treating team. High-lead ambulatory monitoring showed no ventricular ectopy or recurrent arrhythmia after treatment. Cardiac MRI showed preserved biventricular function with non-specific inferior wall findings; possible subtle enhancement was not diagnostic of definite scar, and the findings do not conclusively establish or exclude an acute myocardial process.

An inherited electrical disorder also cannot be definitively excluded. The available material does not provide a reliable QTc from immediately before or during the reported event, a diagnostic Brugada provocation result, or a completed inherited-arrhythmia genetic test result. High-lead monitoring, exercise testing, and adenosine assessment contributed to the inpatient evaluation but cannot individually exclude every intermittent channelopathy. The pathogenic CUL3 variant establishes Gordon's syndrome; it should not be interpreted as evidence of a separate primary arrhythmogenic syndrome.

The decision to implant an ICD requires careful interpretation. Guidance on ventricular arrhythmias emphasises identifying and correcting reversible causes before assessing the need for secondary-prevention ICD therapy [3]. This patient had preserved ventricular function and a potentially reversible electrolyte disturbance. Furthermore, the original rhythm cannot be independently verified now. On the other hand, the treating cardiology and electrophysiology team considered the contemporaneously documented pre-hospital event, uncertainty about its mechanism, and the patient's previous marked hyperkalaemia during interruption of essential treatment when assessing future risk. The ICD was implanted following their multidisciplinary assessment and discussion with the patient. This case-specific decision should not be interpreted as a general indication for ICD implantation in Gordon's syndrome or after every episode associated with hyperkalaemia.

The choice of an extravascular system was separate from the decision to implant an ICD. It avoids chronic transvenous intracardiac leads, a consideration for a relatively young patient who may require long-term device therapy. Anti-tachycardia pacing is principally relevant to selected organised ventricular tachycardias and should not be presented as a reliably effective treatment for torsade de pointes [7].

The principal limitation is the permanently unavailable pre-hospital rhythm recording. It prevents independent assessment of the rhythm diagnosis, preceding QT interval, initiation sequence, and possible artefact. The absence of a reliable contemporaneous QTc and full sampling details for the discordant potassium results further limits mechanistic interpretation. The report's value lies in documenting the clinical decisions and diagnostic uncertainty surrounding a reported serious rhythm episode in a patient with genetically confirmed Gordon's syndrome after interruption of thiazide therapy, rather than establishing a new arrhythmogenic effect of CUL3 or proving that hyperkalaemia caused torsade de pointes.

## Conclusions

This case concerns a patient with genetically confirmed CUL3-related Gordon's syndrome who developed hyperkalaemia after a three-week interruption of bendroflumethiazide. During pre-hospital assessment, ambulance clinicians documented a self-terminating rhythm they identified as torsade de pointes. Because the original recording cannot be retrieved, the rhythm cannot be independently classified, and neither can its mechanism nor a causal relationship with hyperkalaemia be established.

The case highlights the importance of continuity of thiazide therapy in Gordon's syndrome and careful assessment of discordant point-of-care and laboratory potassium results. It also illustrates the need to investigate a reported serious arrhythmia while recognising the limits of the available evidence. The decision to implant an ICD despite a potentially reversible metabolic disturbance was made through individual multidisciplinary assessment and should not be generalised to all patients with Gordon's syndrome.
